# The inspiration of methyltransferase in RNA methylation modification for targeted therapy of malignant tumors

**DOI:** 10.3389/fimmu.2025.1663423

**Published:** 2025-11-03

**Authors:** Xinru Chen, Xinyu Liu, Jiaqi Xu, Xingyu Zhu, Bingyao Liu, Xinhao Yang, Ge Cong, Xiaoyan Lin, Wei Chong, Hao Chen

**Affiliations:** ^1^ Department of Gastrointestinal Surgery, Shandong Provincial Hospital Affiliated to Shandong First Medical University, Jinan, Shandong, China; ^2^ Key Laboratory of Engineering of Shandong Province, Shandong Provincial Hospital, Medical Science and Technology Innovation Center Shandong First Medical University & Shandong Academy of Medical Sciences, Jinan, Shandong, China; ^3^ Department of Pathology, Shandong Provincial Hospital, Shandong University, Jinan, Shandong, China; ^4^ Department of Pathology, Shandong Provincial Hospital Affiliated to Shandong First Medical University, Jinan, Shandong, China; ^5^ Clinical Research Center of Shandong University, Clinical Epidemiology Unit Qilu Hospital of Shandong University, Jinan, Shandong, China

**Keywords:** RNA methylation modification, writer, malignant tumor, mechanism of action, targeted therapy

## Abstract

RNA methylation modification has always been a research hotspot. RNA methylation modification can regulate processes such as transcription, translation, splicing, stability, and degradation of RNA, in which effector proteins play an important role, including ‘writers’, ‘erasers’, and ‘readers’. There are various types of proteins involved in cancer progression, and in recent years, research on their mechanisms of action has been increasing, providing new ideas for targeted cancer therapy. By regulating the expression of related genes and affecting signaling pathways, protein writing plays a role in promoting or inhibiting cancer in the proliferation, invasion, migration, and metastasis of different tumors, providing direction for the treatment of malignant tumors. This article reviews the mechanisms of common RNA methylation modified writers and their prospects in targeted cancer therapy.

## 1 Introduction

**Figure 1:**
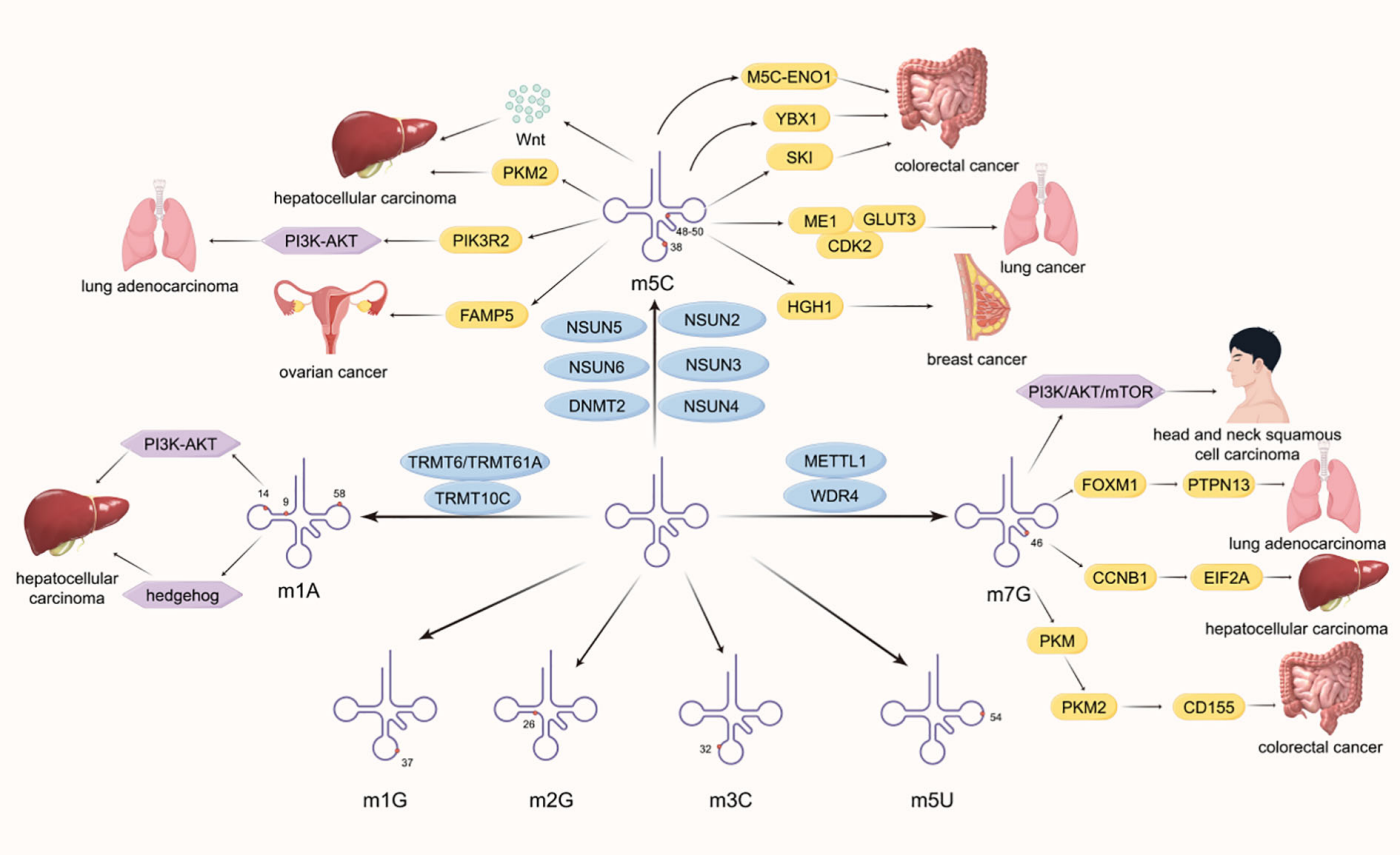
This demonstrates the distribution of different RNA methylation modifications in tRNA and the mechanism and principles of how some methyltransferases affect the development of malignant tumors. Common methylation sites include N1 methyladenosine (m1A), N3 methylcytosine (m3C), N5 methylcytosine (m5C), N1 methylguanosine (m1G), N2 methylguanosine (m2G), and N7 methylguanosine (m7G).

RNA modification was first discovered and recorded in the 1950s, and now over 170 types of RNA modifications have been discovered, which are commonly present in both coding and non-coding RNA. RNA modification is mostly reversible and has functions such as splicing, localization, transport, translation, and degradation, which are crucial for regulating various types of RNA (1, 2). At the cellular level, RNA modification is involved in various cellular processes including cell death, proliferation, aging, differentiation, migration, metabolism, autophagy, DNA damage response, and liquid-liquid phase separation (3). The abnormal expression of RNA modification can serve as a signal for the occurrence of malignant tumors, and is related to the functions of malignant tumor cell growth, proliferation, self-renewal, stress adaptation, invasion, and resistance to therapy. RNA methylation is the most common characteristic modification of RNA modification. According to the methylation sites, RNA methylation can be classified into various forms, including N6-methyladenosine (m6A), 5-methylcytosine (m5C), N1-methyladenosine (m1A), N7-methylguanosine(m7G), N6,2′-O-dimethyladenosine (m6Am), N4-acetylcytosine (ac4C), Pseudouridine (Ψ). N6 methyladenosine (m6A) modification is a common RNA methylation modification that was first discovered in research in the 1970s and has been a hot topic in disease mechanism studies both domestically and internationally in recent years (4, 5). Many studies have shown that m6A plays an important role in human cancer development and tumorigenesis (6, 7). It is mainly related to three regulatory factors, which are the methyltransferase (writers), demethylase (erasers), and binding proteins (readers) (8, 9). Writers, also known as m6A methyltransferase complex, are composed of a variety of components, mainly including methyltransferase-like 3 (METTL3) and methyltransferase‐like 14 (METTL14) with some regulatory subunits such as the Wilms’ tumor 1 (WT1) associated protein (WTAP) (8). METTL3 is a 70 kda protein that is one of the important components of writers and belongs to a conserved family of methyltransferases (10). Structurally, METTL3 contains 580 amino acids in its full length and is composed of a methyltransferase domain(MTD)and a zinc finger domain (ZFD) (11). Besides, it contains S-adenosylmethionine (SAM) to catalyze methyl transfer (12). METTL3 is a monomer with catalytic activity, while METTL14 plays a structural role in RNA substrate recognition (13), and the two can form stable heterodimers (14). The heterodimers are called M6A-METTL Complex (MAC) (15) and formed by the MTDs of both. The solution structure of the ZFD of METTL3 provides methyltransferase activity of METTL3-METTL14 (16). METTL3 (11) and METTL14 (17) play a crucial role in the progression of human malignant tumors and provide new ideas for the treatment of malignant tumors, such as immunotherapy (18). WTAP can interact with METTL3 and METTL14 to regulate the nuclear speckle localization of methyltransferases and their catalytic activity *in vivo (* 19). It can also regulate recruitment of the m6A methyltransferase complex to mRNA targets (19). Based on its multiple functions and its close relationship with the cyclical activity, metabolism and autophagy of tumor cells, WTAP has great potential in clinical cancer treatment and prognosis (20). The methyltransferase complex is also composed of KIAA1429 (17, 21), METTL5, tRNA methyltransferase activator subunit 11-2(TRMT112), Cbl proto-oncogene like 1 (CBLL1), zinc finger protein 217 (ZFP217), RNA binding motif protein 15 (RBM15), RNA binding motif protein 15B (RBM15B), vir-like m6A methyltransferase-associated protein (VIRMA), zinc finger CCCH-type containing 13 (ZC3H13), and methyltransferase-like protein 6 (METTL16) (9). METTL5 can modify 18S rRNA (22) and interact with SAM (23),which is associated with the occurrence of pancreatic cancer (24). ZC3H13 has the effect of inducing the translocation of methylase complexes into the nucleus (25). METTL16 can regulate bone marrow cell differentiation, and the absence of red blood cells can cause apoptosis, which may be associated with malignant hematologic tumors (12). In recent years, researchers have also discovered that ZCCHC4 acts as an m6A methyltransferase to modify 28S rRNA (22) and plays an important role in the translation process (26). 5-methylcytosine (m5C) is also a common RNA methylation modification, widely present in tRNA, mRNAs, rRNAs, and enhancer RNA (eRNA) (27, 28). It involves adding a methyl group to the fifth carbon atom of cytosine, including the seven members of the NOL1/NOP2/Sun domain family (NSUN) NSUN1 to NSUN7 and DNA methyltransferase like 2 (DNMT2) (29). N1 methyladenosine (m1A) is the addition of a methyl group to the nitrogen atom at the first position of adenosine (30, 31), and has been found in transfer RNAs (tRNAs), rRNAs, mRNAs, lncRNAs, and mitochondrial RNAs (32, 33). M1A mainly exists in tRNA and rRNA, first identified in tRNA, usually located at positions 9, 14 and 58 of tRNA (34)(**Figure 1**). The methyltransferase TRMT6/61A present in tRNA recognizes the T-Loop like structure (35). Another methyltransferase responsible for m1A modification in tRNA is tRNA methyltransferase 10 homolog A (TRMT10) (36). M7G is an RNA modification that adds a methyl group to the N7 position of the nucleoside (37, 38), and is widely present in the 5’CAP structure of eukaryotic mRNA and pre tRNA (39). It plays an important role in affecting processing maturation (40), nuclear cytoplasmic transport (41), stability (42), transcription, and translation (43, 44). The methyltransferases responsible for m7G modification are mainly methyltransferase like 1 (METTL1) and its cofactor WD repeat domain 4 (WDR4) (45, 46). M5C, m1A, and m7G modifications can also play a role in the progression of malignant tumors. NSUN2 promotes the progression of lung cancer and hepatocellular carcinoma by affecting cellular metabolism (47, 48). The TRMT6/TRMT61A driving signaling pathway affects the progression of malignant tumors (49, 50). METTL1 and WDR4 promote cancer progression and poor prognosis by synergistically regulating the translation process (42, 51, 52). This review focuses on the mechanism of action of RNA writer in malignant tumors and provides inspiration and assistance for targeted therapy (**Figure 1**).

## 2 RNA methylation modification

More and more studies have shown that RNA methylation modified writers are closely related to the occurrence and development of various malignant tumors (53, 54), but their mechanisms of action varied in different malignant tumors. The following will discuss the mechanisms of action in different malignant tumors and thoughts on targeted therapy.

### 2.1 m6A

#### 2.1.1 METTL3

In recent years, a number of studies have found that METTL3 is involved in cancer development as a cancer promoting factor. METTL3 can promote the proliferation and metastasis of esophageal cancer cells (55) and renal cell carcinoma (RCC) cells (56). In addition, researchers have provided a new direction for cancer treatment through continuous exploration of its mechanism(**Table 1**). EPPK1 is a gene which inhabits the proliferation of esophageal cells obviously. METTL3, as a m6A writer, its deficiency can down-regulate the expression of EPPK1 and thereby inhibit the PI3K/AKT pathway, then curbing the development of esophageal cancer (57). METTL3 promotes cancer stem-like cell (CSC) phenotype in oral squamous cell carcinoma(OSCC) by targeting SALL4 through the Wnt/β-catenin signaling pathway (58). METTL3 mediates miR-99a-5p to drive OSCC cell metastasis through targeting ZBTB7A (59). METTL3 can also promote the occurrence of OSCC via m6A modification-dependent stabilization of LAMA3 transcripts (60). There are many kinds of lncRNAs, including LINC00894 (61), HOXA10-AS (62) and more. In papillary thyroid carcinoma (PTC), METTL3 can improve and enhance the stability of LINC00894 and thus promote the development of PTC through the m6A-YTHDC2-dependent pathway (61). METTL3 can modify lncRNA DSCAM-AS1 to enhance the stability of endogenous antioxidant factor SLC7A11, thereby inhibiting iron death and promoting the poor prognosis of breast cancer (63). In addition, METTL3 can promote the m6A modification of HOXA10-AS and enhance its RNA stability, thereby driving the malignant progression of laryngeal squamous cell carcinoma (LSCC) (62). LncRNA and related genes and their signaling pathways may be effective mechanistic targets for cancer treatment targeting METTL3. MicroRNAs also play a role in tumorigenesis. MiR-1908-5p is a type of microRNA that plays a role in the progression of nasopharyngeal carcinoma (NPC). METTL3 mediates the m6A modification of miR-1908-5p by targeting HOPX, thereby promoting the occurrence of NPC (64). METTL3 is involved in the m6A modification of the nuclear protein DEK, which improves the stability and expression of DEK, thereby promoting the proliferation and migration of gastric cancer (GC) cells (65). METTL3 mediates STAT5A regulation of KLF4 to promote proliferation and migration of GC cells (66). METTL3 promotes the development of ovarian cancer (OC) by increasing the mRNA level of oncogene MALAT1 through RNA binding protein ELAVL1 (67). METTL3 mediates HSPA9 m6A modification to promote the malignant development of cervical cancer (CC) (68). In another mechanism, METTL3 promotes the malignant progression of CC by up-regulating the expression of NEK2 (69). METTL3 can also enhance the stability of the glutamine transporter SLC38A1 mRNA, drive the growth of CC cells, and inhibit their apoptosis (70). METTL3 mediates Ran GTPase activating protein 1 (RanGAP1) to contribute to the progress of colorectal cancer (CRC) (71). METTL3 can promote the senescence of CRC cells through the mediator CDKN2B, providing a new target for the treatment of CRC (72). METTL3 mediated m6A modification of matrix metalloproteinase-9 (MMP9) mRNA promotes tumorigenicity of CRC (73). METTL3 can mediate the expression of circular RNA circ_0008345, which can bind to miR-182-5p and prevent miR-182-5p from inhibiting the oncogene CYP1A2, thereby contributing to the malignant progression of CRC (74). Meanwhile, METTL3 promotes epithelial-mesenchymal transition in CRC through m6A modification of SNAIL mRNA, where SNAIL enhances CXCL2 secretion through the NF-κB pathway to recruit M2 macrophages and promote CRC lung metastasis (75). METTL3 mediates m6A modification of exonuclease DCLRE1B to promote the development of pancreatic cancer (PC) (76). METTL3 induces m6A modification of FBXO31 and enhances its mRNA translation, thereby promoting the occurrence of PC (77). METTL3 mediates MiR‐589‐5p maturation and expression, which is positively correlated and over-expression can promote the deterioration of liver cancer (78). METTL3 promotes the proliferation and stemness of liver cancer stem cells (LCSCs) by targeting SOCS3 mRNA through the JAK2/STAT3 pathway (79). METTL3 mediates m6A modification of lnc-TSPAN12 to drive migration and invasion of hepatocellular carcinoma(HCC) cells, which in turn promotes hepatic metastasis (80). The transcription factor RELA can bind to the METTL3 promoter region to promote the transcription of METTL3, thereby promoting the malignant growth of HCC cells (81). Another circular RNA also plays a role in METTL13’s involvement in tumor progression. In prostate cancer (PCa), METTL3 induces homeobox C6 (HOXC6) m6A modification to stabilize its expression and promote cancer cell proliferation, invasion, migration, stemness, and glycolysis (82). METTL3 mediates CircGlis3 (HSA_CIRC_0002874) to up-regulate MDM2 and regulate the p53 signaling pathway, promoting PCa cell proliferation, migration, and invasion (83). Aromatase (CYP19A1) is an enzyme involved in limiting the rate of estrogen biosynthesis. METTL3 promotes estrogen production by enhancing CYP19A1 translation and mediates miR-196a, thereby driving non-small cell lung cancer (NSCLC) metastasis (84). In another mechanism, METTL3 promotes the occurrence and metastasis of NSCLC by inhibiting FDX1 by promoting the maturation of miR-21-5p (85). Due to different mechanisms, METTL3 can also play different roles in the same cancer. The over-expression of SH3 domain binding protein 5 (SH3BP5) can inhibit the invasion ability of lung cancer cells, METTL3 can mediate m6A modification to promote its expression, and METTL3 itself also has the function of inhibiting the migration and invasion of lung cancer cells, thereby METTL3 can inhibit the deterioration of lung cancer (86). METTL3 inhibits malignant progression of lung adenocarcinoma by blocking the PI3K/AKT/NF-κB signaling pathway (87). METTL3 exhibits a dual role in cancer, highlighting its highly environmentally dependent function. This contradictory phenomenon may stem from METTL3’s ability to modify various RNA substrates, including transcripts of oncogenes and tumor suppressor genes. The ultimate effect is likely to be the result of the combined effects of specific cellular backgrounds, tumor microenvironments, and transcriptome landscapes. This reminds us that strict biomarker stratification is necessary when applying METTL3 inhibitors in clinical practice to achieve precise treatment (**Table 1**).

**Table 1:** The mechanism of METTL3 in malignant tumors.

Malignant tumors types	Roles	Mechanism(target/signaling pathway)	References
esophageal cancer	tumor promoter	EPPK1/PI3K/AKT	(57)
OSCC	tumor promoter	SALL4/Wnt/β-catenin	(58)
OSCC	tumor promoter	miR-99a-5p/ZBTB7A	(59)
OSCC	tumor promoter	LAMA3	(60)
PTC	tumor promoter	LINC00894/m6A-YTHDC2-dependent	(61)
LSCC	tumor promoter	HOXA10-AS	(62)
breast cancer	tumor promoter	DSCAM-AS1/SLC7A11	(63)
NPC	tumor promoter	miR-1908-5p/HOPX	(64)
GC	tumor promoter	DEK	(65)
GC	tumor promoter	STAT5A/KLF4	(66)
OC	tumor promoter	MALAT1/ELAVL1	(67)
CC	tumor promoter	HSPA9	(68)
CC	tumor promoter	NEK2	(69)
CC	tumor promoter	SLC38A1	(70)
CRC	tumor promoter	RanGAP1	(71)
CRC	tumor promoter	CDKN2B	(72)
CRC	tumor promoter	MMP9	(73)
CRC	tumor promoter	miR-182-5p/CYP1A2	(74)
CRC	tumor promoter	SNAIL/CXCL2/NF-κB/M2	(75)
PC	tumor promoter	DCLRE1B	(76)
PC	tumor promoter	FBXO31	(77)
liver cancer	tumor promoter	miR‐589‐5p	(78)
liver cancer	tumor promoter	SOCS3/JAK2/STAT3	(79)
HCC	tumor promoter	lnc-TSPAN12	(80)
HCC	tumor promoter	RELA	(81)
PCa	tumor promoter	HOXC6	(82)
PCa	tumor promoter	CircGlis3/MDM2/p53	(83)
NSCLC	tumor promoter	CYP19A1/miR-196a	(84)
NSCLC	tumor promoter	FDX1/miR-21-5p	(85)
lung cancer	tumor suppressor	SH3BP5	(86)
lung cancer	tumor suppressor	PI3K/AKT/NF-κB	(87)

#### 2.1.2 METTL14

METTL14, as one of the main members of m6A writers, plays an important role in the mechanisms related to malignant tumors(**Table 2**). METTL14 enhances the m6A modification of CDKN2A, inhibits the activation of p53 pathway, and promotes retinoblastoma (RB) progression (88). In nasopharyngeal carcinoma, METTL14 promotes cancer progression by regulating the stability of AOC1 mRNA (89). METTL14 catalyzes m6A modification of ANKRD22 mRNA to improve mRNA stability and translation efficiency. At the same time, ANKRD22 interacts with SLC25A1 to increase intracellular acetyl-CoA content, promoting lipid metabolism reprogramming and cellular lipid synthesis, thereby advancing the progression of nasopharyngeal carcinoma (90). METTL14 can up-regulate the expression of lncRNA MSTRG.292666.16 and enhance the level of m6A modification, which can promote the development of NSCLC (91). In another mechanism, METTL14 increases miR-93-5p expression and matures pri-miR-93-5p through m6A alteration to target and inhibit TXNIP, thereby inhibiting NSCLC cell apoptosis and promoting cancer development and metastasis (92). Moreover, METTL14 can target CSF1R to accelerate the proliferation, migration, and invasion of NSCLC cells (93). METTL14 activates miR-29c-3p through m6A and regulates the ubiquitination of pyruvate kinase isoform M2 (PKM2) mediated by the tripartite motif containing 9 (TRIM9), driving aerobic glycolysis of glucose and promoting the progression of triple-negative breast cancer (TNBC) (94). METTL14 mediates lncRNA RP1-228H13.5 to promote the development of liver cancer through targeting hsa-miR-205 and regulating the expression of zinc finger protein interacting with K protein 1 (ZIK1) (95). In glioma cells, downregulation of METTL14 induces down-regulation of m6A modification and expression of circRNA_103239, driving up-regulation of miR-182-5p and down-regulation of MTSS1, thereby promoting EMT in glioma and promoting tumor progression (96). METTL14 targets downstream gene MN1 to enhance its stability and translation efficiency, thereby promoting malignant progression of OC (97). METTL14, by forming the METTL3-METTL14 complex, enhances the m6A modification of SETBP1 mRNA, thereby increasing the stability of SETBP1 mRNA and subsequently activating the PI3K-AKT signaling pathway to promote cell proliferation in myelodysplastic tumors (98). In addition, METTL14 also plays an equally important role in inhibiting the progression of malignant tumors. METTL14 promotes autophagy in OSCC cells and inhibits its progression by targeting the autophagy related gene RB1CC1 through m6A modification (99). METTL14 mediates miR-30c-1-3p maturation, and miR-30c-1-3p targets MARCKSL1 to inhibit its expression, thereby suppressing the development of lung cancer through this mechanism (100). METTL14 up-regulates the m6A level of lnc-PLCB1 to enhance its stability and inhibit the migration and invasion of GC cells (101). METTL14 promotes the degradation of ATF5 mRNA, reduces WDR74 transcription and β - catenin nuclear translocation, thereby inhibiting GC cell stemness and slowing down cancer progression (102). In colon cancer, METTL14 targets SCD1, and its over-expression significantly enhances the m6A modification of SCD1 mRNA and reduces SCD1 mRNA levels, possibly through SCD1 mediated Wnt/β- Catenin signaling inhibits the stemness and metastasis of colon cancer cells, thereby hindering the tumorigenic process of colon cancer (103). METTL14 inhibits the expression of transcription factors NANOG and β-catenin in CRC cells, thereby inhibiting the phenotype of CRC stem cells and hindering cancer progression (104). METTL14 targets FTH1 to reduce its mRNA stability, thereby enhancing sorafenib induced ferroptosis and helping to inhibit CC progression through the PI3K/Akt signaling pathway (105). Similar to METTL3, METTL14 also exhibits dual functionality. The role of METTL14 may be related to its cooperative relationship with METTL3 and other co factors. When dissociated from METTL3, METTL14 may be more inclined to stabilize tumor suppressor RNA. This suggests that targeting the METTL3-METTL14 interaction interface may be a more precise strategy than comprehensive inhibition (**Table 2**).

**Table 2:** The mechanism of METTL14 in malignant tumors.

Malignant tumors types	Roles	Mechanism(target/signaling pathway)	References
RB	tumor promoter	CDKN2A/p53	(88)
nasopharyngeal carcinoma	tumor promoter	AOC1	(89)
nasopharyngeal carcinoma	tumor promoter	ANKRD22/SLC25A1	(90)
NSCLC	tumor promoter	MSTRG.292666.16	(91)
NSCLC	tumor promoter	miR-93-5p/TXNIP	(92)
NSCLC	tumor promoter	CSF1R	(93)
TNBC	tumor promoter	miR-29c-3p/TRIM9/PKM2	(94)
liver cancer	tumor promoter	RP1-228H13.5/hsa-miR-205/ZIK1	(95)
glioma	tumor promoter	circRNA_103239/miR-182-5p/MTSS1	(96)
OC	tumor promoter	MN1	(97)
myelodysplastic tumor	tumor promoter	SETBP1/PI3K/AKT	(97)
OSCC	tumor suppressor	RB1CC1	(99)
lung cancer	tumor suppressor	miR-30c-1-3p/MARCKSL1	(100)
GC	tumor suppressor	lnc-PLCB1	(101)
GC	tumor suppressor	ATF5/WDR74/β - catenin	(102)
colon cancer	tumor suppressor	SCD1/Wnt/β- Catenin	(103)
CRC	tumor suppressor	NANOG/β - catenin	(104)
CC	tumor suppressor	FTH1/PI3K/Akt	(105)

#### 2.1.3 WTAP

**Figure 2:**
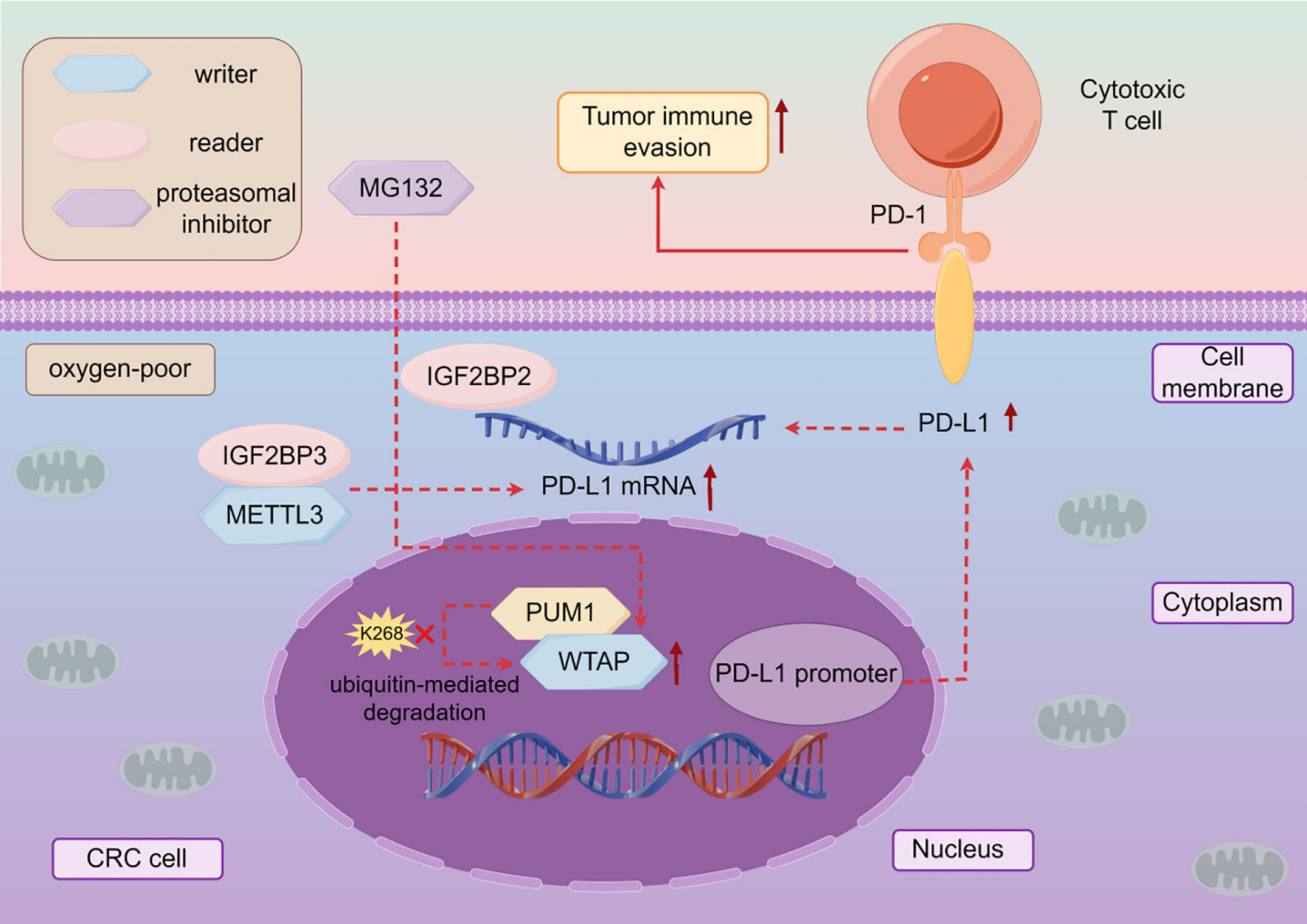
The mechanism of action of WTAP in cancer immunotherapy. In CRC, hypoxia can increase the expression of WTAP, and PUM1 can directly bind to WTAP to inhibit ubiquitin mediated degradation of WTAP under hypoxic conditions, thereby enhancing the expression of PD-L1 and the stability of IGF2BP2 and PD-L1 mRNA binding, inhibiting the anti-tumor activity of T cells, in other words, promoting cancer progression.

The high expression of WTAP is closely related to the poor prognosis caused by many malignant tumors such as NSCLC (106),CRC (107). Therefore, targeted therapy research can be conducted based on the development mechanism of WTAP in different malignant tumors (**Table 3**). WTAP increases the stability of plasminogen activator urokinase (PLAU) and promotes the migration, invasion, and proliferation of LSCC cells (108). WTAP positively regulates the expression of m6A target PTP4A1, activates the AKT mTOR pathway, and promotes esophageal squamous cell carcinoma (ESCC) cell proliferation (109). WTAP can also promote the growth and metastasis of ESCC tumors by reducing the expression of CPSF4 (110). WTAP can affect the progression of malignant tumors by mediating circular RNAs (circRNAs). WTAP mediates circEEF2 to regulate the expression of CANT1 and promote the occurrence of lung adenocarcinoma (LUAD) (111). WTAP can promote the expression of NUPR1, eliminate the role of WTAP knockdown in promoting iron death and inhibiting the malignant behavior of TNBC cells, while up-regulating LCN2 can inhibit iron death and promote the progress of TNBC (112). WTAP mediated FAM83H-AS1 promotes GC cell migration, proliferation, and invasion through m6A modification, thereby promoting cancer development (113). The Warburg effect is an abnormal energy metabolism in which cancer cells obtain energy through oxidative glycolysis, thereby promoting proliferation and cancer progression (114, 115). WTAP can enhance the stability of HK2, accelerate the Warburg effect of GC, and promote GC progression (116). WTAP can also accelerate the migration and epithelial mesenchymal transition of GC cells by promoting the expression of transforming growth factor-beta (TGF-β) (117). WTAP inhibits downstream gene FLNA, enhances the proliferation of colon cancer cells, and inhibits cancer cell autophagy (118). Meanwhile, WTAP mediates downstream molecule VEGFA activation of MAPK signaling, accelerating CRC cell proliferation, migration, invasion, and angiogenesis (119). WTAP also mediates PDK4 and inhibits its expression to drive the proliferation, migration, and invasion of CRC cells (120). In addition, WTAP has also become an important target and research direction in immunotherapy for tumors. PD-L1 (121), as an immunosuppressive checkpoint, can promote immune escape, while WTAP can enhance its expression, strengthen the immune escape mechanism, prevent the anti-tumor effect caused by T cell proliferation (122), and promote the progression of CRC (123) (**Figure 2**). The decreased expression of the tumor suppressor gene PTEN leads to cellular dysregulation and loss, thereby promoting the characteristics of endometrial cancer (EC) stem cells. The reason for this is that down-regulation of WTAP reduces m6A modification of EGR1 mRNA and lowers EGR1 levels (124). Moreover, WTAP can also down-regulate the expression of CAV-1, activate the nuclear factor kappa B (NF-κB) signaling pathway in EC cells, promote EC cell proliferation, migration, and invasion, and accelerate cancer progression (125). WTAP can increase the stability of GBE1 mRNA, and over-expression of GBE1 promotes PC cell proliferation and stemness-like properties (126). Overexpression of WTAP can counteract the tumor suppressive effect of miR-455-3p in PCa cells, thereby promoting PCa progression (127). WTAP can target NRF2 to inhibit iron death and accelerate the malignant progression of bladder cancer(BLCA) (128). In addition, the mRNA of WTAP can interact with long non-coding RNAs (lncRNA) to promote the migratory progression of malignant tumors, such as breast cancer (129). WTAP can also target lncRNA DIAPH1-AS1 to enhance its stability and promote the progression of NPC (130). In renal cell carcinoma (RCC), WTAP regulates the m6A modification of long non coding RNA TEX41 to promote cancer cell proliferation and metastasis (131). WTAP often promotes cancer progression through mechanisms involving immune escape, metabolic reprogramming, and RNA stability. The stabilizing effect of WTAP on PD-L1 mRNA under hypoxic conditions reveals a noteworthy association between m6A modification and immune therapy resistance. Targeting WTAP may enhance the efficacy of immune checkpoint blockade therapy, especially in hypoxic tumors (**Figure 2**, **Table 3**).

**Table 3:** The mechanism of WTAP in malignant tumors.

Malignant tumors types	Roles	Mechanism(target/signaling pathway)	References
LSCC	tumor promoter	PLAU	(108)
ESCC	tumor promoter	PTP4A1/AKT mTOR	(109)
ESCC	tumor promoter	CPSF4	(110)
LUAD	tumor promoter	circEEF2/CANT1	(111)
TNBC	tumor promoter	NUPR1/LCN2	(112)
GC	tumor promoter	FAM83H-AS1	(113)
GC	tumor promoter	HK2	(116)
GC	tumor promoter	TGF-β	(117)
colon cancer	tumor promoter	FLNA	(118)
CRC	tumor promoter	VEGFA/MAPK	(119)
CRC	tumor promoter	PDK4	(120)
CRC	tumor promoter	PD-L1	(123)
EC	tumor promoter	EGR1/PTEN	(124)
EC	tumor promoter	CAV-1/NF-κB	(125)
PC	tumor promoter	GBE1	(126)
PCa	tumor promoter	miR-455-3p	(127)
BLCA	tumor promoter	NRF2	(128)
NPC	tumor promoter	DIAPH1-AS1	(130)
RCC	tumor promoter	TEX41	(131)

#### 2.1.4 METTL16

Current research indicates that METTL16 plays a dual role in cancer biology, either promoting or inhibiting tumor progression depending on the context. In lung cancer, METTL16 interacts with eIF4E2 to enhance the translation of key oncogenes, thereby driving tumor development (132). In breast cancer, METTL16 regulates GPX4 expression, inhibiting ferroptosis and promoting cancer cell proliferation (133). In HCC, METTL16 targets the eukaryotic translation initiation factor 3 subunit a (eIF3a) to promote rRNA maturation and enhance mRNA translation efficiency, thereby facilitating the self-renewal and carcinogenesis of liver cancer stem cells (134). Additionally, METTL16 regulates the stability of SENP3 mRNA, with which it is positively correlated, and promotes the malignant progression of HCC through the METTL16-SENP3-LTF signaling axis by modulating ferroptosis (135). In lung cancer metastasis, METTL16 regulates the stability of SYNPO2L, mediates the secretion of COL10A1 by cancer-associated fibroblasts (CAFs), and promotes epithelial-mesenchymal transition (EMT), making tumor cells more prone to metastasis (136). Furthermore, METTL16 is implicated in metal carcinogenesis; it targets glutamate-ammonia ligase (GLUL) to regulate glutamine metabolism, thereby promoting chromium (VI) exposure-induced tumorigenesis (137). In osteosarcoma, METTL16 targets vacuolar protein sorting protein 33b (VPS33B) for degradation, driving tumor progression (138). In CRC, METTL16 up-regulates pyruvate dehydrogenase kinase 4 (PDK4) by targeting suppressor of glucose by autophagy (SOGA1), thereby promoting CRC progression (139). In acute myeloid leukemia (AML), METTL16 drives tumor development by promoting the expression of branched-chain amino acid (BCAA) transaminases BCAT1 and BCAT2, reprogramming BCAA metabolism (140). In cholangiocarcinoma (CCA), METTL16 targets PRDM15 to regulate FGFR4 expression, promoting cell proliferation and malignant progression (141). These mechanisms collectively highlight the tumor-promoting role of METTL16 in various cancers. Conversely, METTL16 also exhibits tumor-suppressive functions in certain contexts. In PTC, METTL16 increases m6A abundance in SCD1, activates lipid metabolism, and inhibits tumor progression (142). In BLCA, METTL16 reduces the stability of prostate transmembrane protein androgen-induced 1 (PMEPA1) mRNA, inhibiting cell proliferation and cisplatin chemoresistance, thus offering a potential therapeutic strategy for BLCA (143). In epithelial ovarian cancer (EOC), METTL16 negatively correlates with lncRNA MALAT1, and their interaction up-regulates β-catenin, thereby inhibiting the growth, migration, and invasion of EOC cells (144). These findings underscore the dual role of METTL16 in cancer biology and provide valuable insights for clinical interventions and targeted cancer therapies. METTL16 is emerging as an environmentally dependent regulatory factor, playing a role in translation, metabolism, and ferroptosis. It participates in rRNA methylation, which extends the research scope of m6A beyond mRNA. The effect of METTL16 on ferroptosis suggests that it may regulate therapeutic responses, particularly to drugs that induce oxidative stress.

#### 2.1.5 RBM15

RBM15 plays a pivotal role in the progression of various cancers through diverse molecular mechanisms. In triple-negative breast cancer, RBM15 facilitates tumor growth by enhancing serine and glycine metabolism (145). In LSCC, RBM15 mediates m6A modification of TMBIM6 mRNA, increasing its stability and contributing to poor prognosis (146). In CRC, RBM15 binds to E2F2 and stabilizes its mRNA, thereby promoting malignant cellular processes (147). In CC, RBM15 drives m6A modification and protein translation of EZH2, leading to enhanced cancer cell proliferation, invasiveness, and epithelial-mesenchymal transition (EMT) (148). Additionally, RBM15 stabilizes the expression of the oncogenic lncRNA HEIH, promoting the proliferation, metastasis, and stemness of CC cells (149). In NSCLC, RBM15 up-regulates Kruppel-like factor 1 (KLF1), which suppresses the expression of TRIM13, a member of the tripartite motif (TRIM) family, and promotes ANXA8, a member of the annexin A (ANXA) family, thereby accelerating cancer progression (150). In LUAD, RBM15 enhances the stability of LDHA mRNA, further supporting tumor progression (151). Moreover, RBM15 up-regulates TGF-β/Smad2 expression, promoting the growth, invasion, and migration of lung cancer cells (152). In CC, RBM15 activates the AKT/mTOR signaling pathway to promote the expression of the oncogene OTUB2, driving malignant progression (153). Conversely, knockdown of RBM15 suppresses CC tumorigenesis by inhibiting the JAK-STAT pathway (154). In paclitaxel resistant ovarian cancer, silencing RBM15 down-regulates multidrug resistance 1 (MDR1) expression and inhibits malignant progression (155). Furthermore, RBM15-mediated m6A modification of MyD88 mRNA promotes CRC occurrence and metastasis, while its knockdown inhibits these processes (156). In clear cell renal cell carcinoma (ccRCC), RBM15 stabilizes CXCL11 mRNA, enhancing cell proliferation, colony formation, macrophage infiltration, and EMT (157). Interestingly, RBM15 exhibits a contrasting role in certain cancers. For instance, its elimination inhibits PC progression and enhances macrophage infiltration and phagocytosis, offering a novel direction for PC immunotherapy (158). The above mechanism suggests that RBM15 may serve as a “core stabilizer” for malignant tumors, generating diverse phenotypes in different cancer backgrounds by regulating the basic mechanism of transcriptional stability. Targeting RBM15 may simultaneously disrupt the energy supply and biosynthetic ability of tumors. In addition, the regulation of tumor immune microenvironment by RBM15 is highly environment dependent, and future immune combination strategies require precise tumor typing guidance. RBM15 inhibitors may have a synergistic effect with traditional chemotherapy drugs and serve as an important strategy for future clinical treatment.

#### 2.1.6 KIAA1429

KIAA1429 plays a significant role in cancer progression through its regulation of m6A modification and mRNA stability. In nasopharyngeal carcinoma, KIAA1429 mediates m6A modification of PTGS2, enhancing its mRNA stability and promoting cancer cell growth, proliferation, migration, and invasion (159). In LUAD, KIAA1429 increases m6A levels in ARHGAP30 mRNA, thereby driving tumor proliferation and metastasis (160). Additionally, KIAA1429 mediates LINC01106 to enhance its expression, increasing phosphorylated JAK2 and STAT3 levels, which further promotes LUAD development (161). KIAA1429 also up-regulates BTG2 expression and enhances its stability, contributing to LUAD progression (162). Furthermore, KIAA1429 promotes LUAD cell proliferation, migration, and invasion by up-regulating MUC3A expression (163). In GC, KIAA1429 down-regulates RASD1 expression by destabilizing its mRNA, thereby facilitating tumor growth and metastasis (164). KIAA1429 also enhances GC resistance to oxaliplatin (OXA) chemotherapy by targeting FOXM1 and stabilizing its mRNA (165). Moreover, KIAA1429 induces LINC00958 to promote aerobic glycolysis in GC cells, leading to poor prognosis (166). In HCC, KIAA1429 inhibits the expression of the tumor suppressor RND3, exerting oncogenic effects and promoting metastasis (167). Additionally, KIAA1429 targets HK1 to regulate the Warburg effect, reducing the sensitivity of liver cancer cells to the chemotherapy drug sorafenib (168). In CRC, KIAA1429 regulates lncRNA POU6F2-AS1 to promote malignant behavior (169) and reduces WEE1 stability, inhibiting its expression and driving CRC proliferation and progression (170). In colon adenocarcinoma (COAD), KIAA1429 activates the HIF-1 signaling pathway to promote tumor development (171). In ovarian cancer, KIAA1429 enhances ENO1 mRNA stability, promoting tumor progression and aerobic glycolysis (172). Finally, in multiple myeloma, KIAA1429 increases FOXM1 expression and stabilizes its mRNA, driving aerobic glycolysis and malignant behavior (173). From the above research, it can be seen that unlike some m6A writers with dual roles, KIAA1429 plays a strong pro cancer role in various types of cancer. It participates in almost all known malignant features of tumors, including proliferation, metastasis, metabolic reprogramming, and chemotherapy resistance, by regulating different downstream targets.

#### 2.1.7 Others

**Figure 3:**
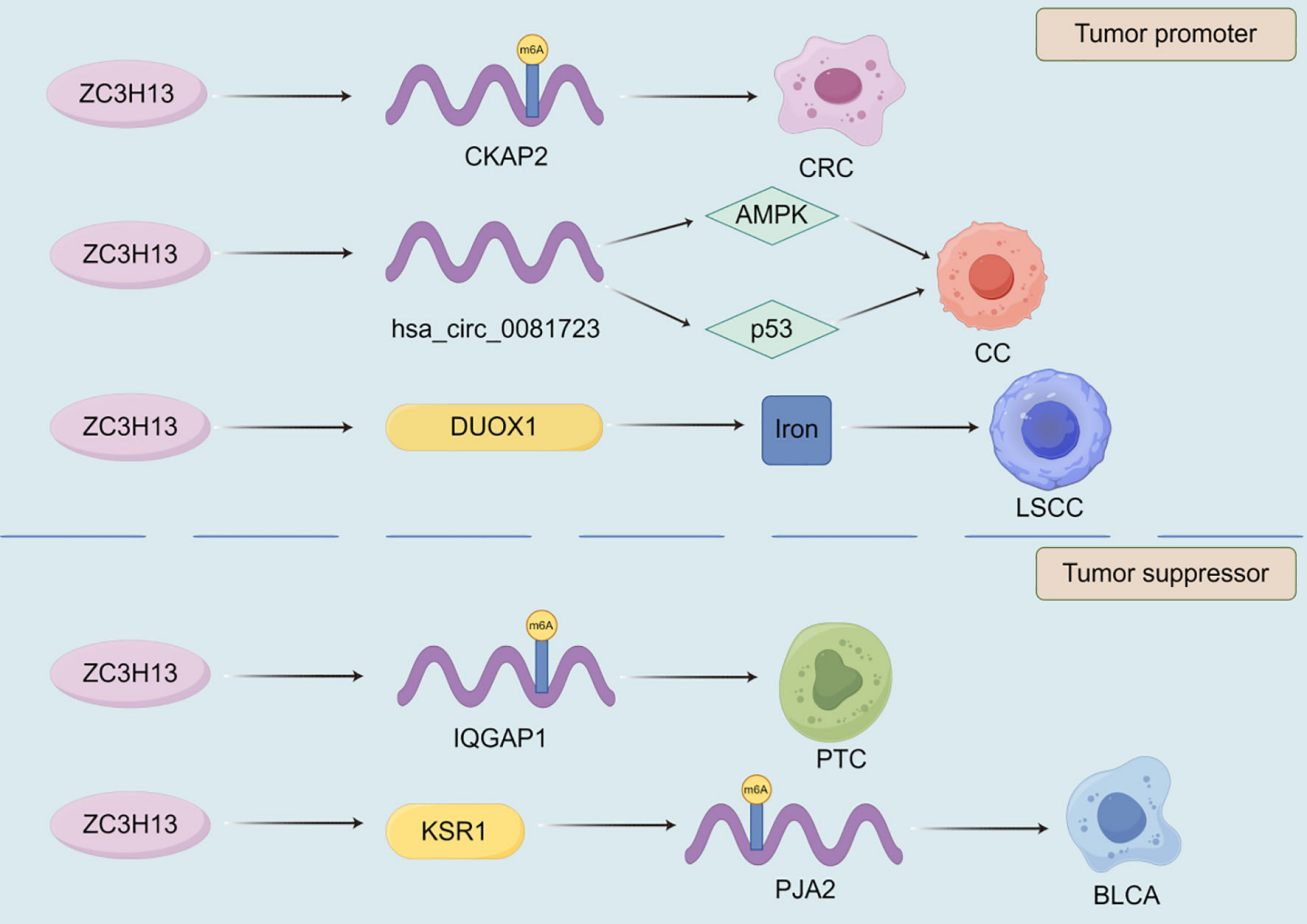
The role of ZC3H13 in the progression of malignant tumors. Among many mechanisms, ZC3H13 can promote or inhibit the progression of malignant tumors by affecting the targeted gene m6A modification.

**Figure 4:**
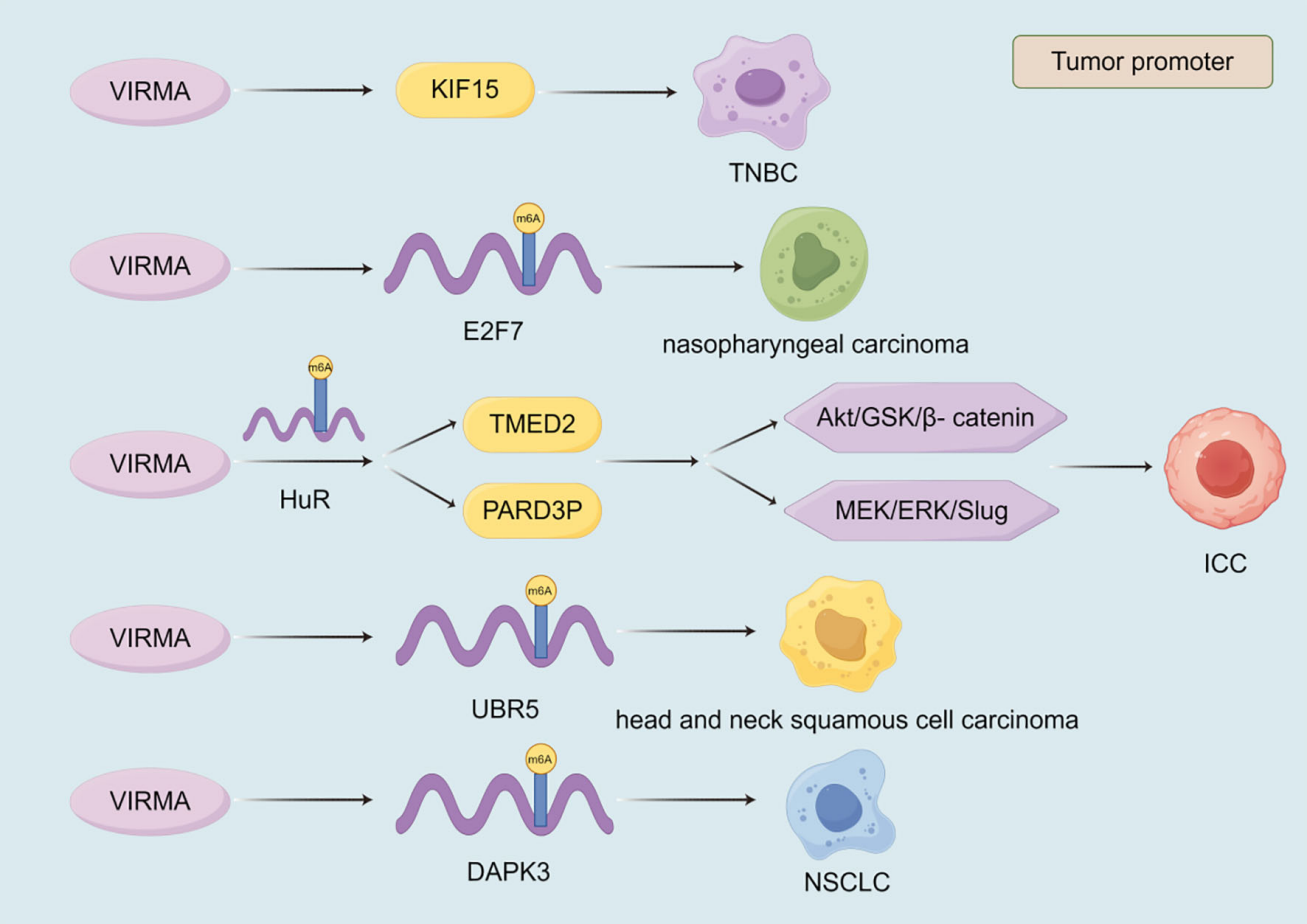
The role of VIRMA in the progression of malignant tumors. The currently discovered roles of VIRMA in malignant tumors are all promoting. VIRMA promotes the progression of malignant tumors by regulating the expression of target genes, the stability of their transcripts, and signaling pathways.

The expression of zinc finger CCCH-type containing 13 (ZC3H13) plays a crucial role in the development of malignant tumor (**Figure 3**). ZC3H13 promotes the malignant progression of CC by mediating CKAP2 m6A modification (174). ZC3H13 mediated circRNA hsa_circ_0081723 m6A modification promotes CC progression by regulating the AMPK/p53 pathway (175). ZC3H13 reverses the increase in iron content in LSCC cells by inhibiting the double oxidase 1 (DUOX1) gene, thereby reducing iron death (176). And, ZC3H13 can mediate m6A modification of centromere protein K (CENPK) to promote the progression of cervical cancer (177). In addition to promoting the development of malignant tumors, ZC3H13 also has inhibitory effects on them. ZC3H13 can inhibit the migration and invasion of hepatocellular carcinoma cells (178). ZC3H13 inhibits the proliferation, invasion, and migration of PTC cells by promoting the degradation of IQGAP1 mRNA (179). ZC3H13 can degrade KSR1, increase the stability of PJA2 mRNA, promote autophagy in BLCA cells, and inhibit BLCA progression (180). As one of the m6A writers, VIRMA also affects the progression of malignant tumors (**Figure 4**). VIRMA promotes the progression of TNBC by up regulating the expression of KIF15 (181). VIRMA can up-regulate the expression of E2F7 and maintain the stability of E2F7 mRNA, promoting the occurrence and metastasis of nasopharyngeal carcinoma (182). VIRMA can maintain the stability of TMED2 and PARD3B through m6A HuR mediation, activate the Akt/GSK/β- catenin and MEK/ERK/Slug signaling pathways, thereby promoting further deterioration of intrahepatic cholangiocarcinoma (ICC) (183). VIRMA promotes the progression of head and neck squamous cell carcinoma by upregulating the m6A level of UBR5 (184). VIRMA promotes NSCLC cell proliferation and malignant progression through m6A dependent degradation of DAPK3 mRNA (185). The mechanisms of these relatively less studied writers often involve the stability of lncRNA/circRNA, metabolic reprogramming, and immune regulation, often exhibiting pro cancer effects, but there are exceptions. They participate in stress adaptation, whether it is metabolic, oxidative, or immune related stress, making them potential targets for combination therapies aimed at disrupting tumor tolerance (**Figures 3**, **4**).

### 2.2 m5C

#### 2.2.1 NSUN2

**Figure 5:**
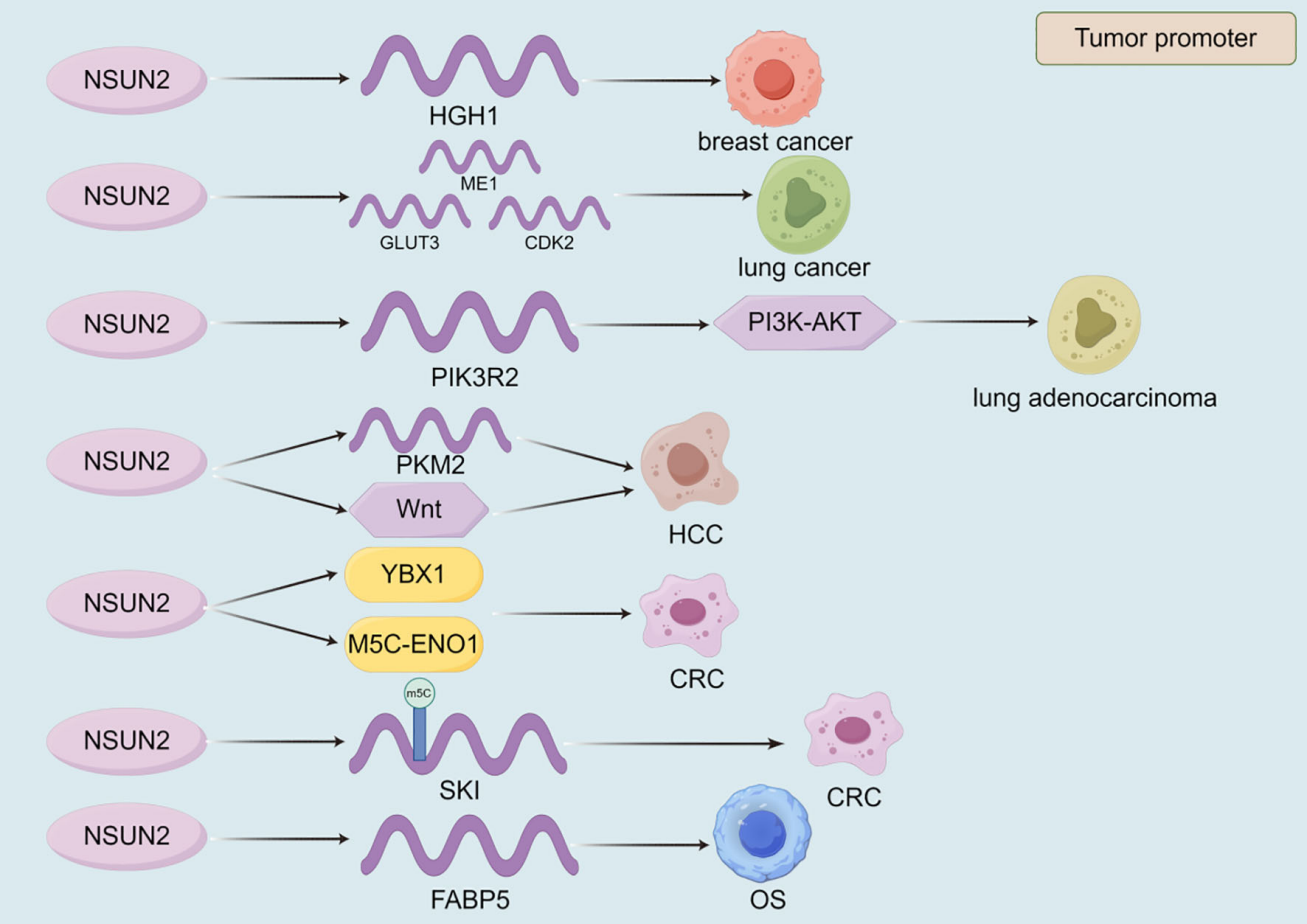
The role of NSUN2 in the progression of malignant tumors. The currently discovered roles of NSUN2 in malignant tumors are all promoting. VIRMA promotes the progression of malignant tumors by regulating the expression of target genes, the stability of their transcripts, and signaling pathways.

NSUN2 plays a pivotal role in the development and progression of various cancers by regulating gene expression and enhancing mRNA stability, serving as a key hub connecting m5C modification with tumor malignancy phenotype (**Table 4**, **Figure 5**). In breast cancer, NSUN2 promotes tumorigenesis by modulating the expression of HGH1 and stabilizing its mRNA, suggesting that HGH1 could be a potential therapeutic target (186). Similarly, in lung cancer, NSUN2 facilitates metabolic reprogramming and drives Cr(VI)-induced malignant transformation by stabilizing the mRNA of key genes such as ME1, GLUT3, and CDK2 (47). Furthermore, NSUN2 up-regulates PIK3R2 and enhances its mRNA stability, leading to the activation of the PI3K-AKT signaling pathway and promoting the malignant progression of lung adenocarcinoma, highlighting its potential as a therapeutic target (187). In HCC, NSUN2 contributes to tumor progression by up-regulating PKM2 and stabilizing its mRNA, thereby promoting glycolysis and offering potential therapeutic avenues for HCC patients (48). Additionally, NSUN2 regulates the Wnt signaling pathway to enhance HCC cell proliferation and migration (188). Notably, Niu et al. discovered that NSUN2 lactylation drives drug resistance in cancer cells, suggesting that targeting NSUN2 lactylation could be an effective strategy to improve patient outcomes (189). In CRC, NSUN2 exerts its oncogenic effects through the NSUN2/YBX1/m5C-ENO1 signaling axis, providing a rationale for combining NSUN2 inhibitors in CRC treatment (190). Moreover, NSUN2 induces m5C modification of SKIL, enhancing its mRNA stability and promoting CRC progression (191). In OS, NSUN2 up-regulates FABP5 expression and stabilizes its mRNA, thereby promoting fatty acid metabolism in OS cells and driving tumor progression (192). These mechanisms indicate that NSUN2 acts as a metabolic core regulator, reshaping glycolysis and fatty acid metabolism by stabilizing key metabolic enzymes such as PKM2 and ENO1 mRNA. NSUN2 also mediates the malignant transformation induced by environmental carcinogen Cr (VI), revealing its special significance in the occurrence of environment related tumors. These findings suggest its enormous potential as a combination therapy target, but highly selective inhibitors need to be developed to overcome the potential toxicity caused by its underlying physiological functions (**Figure 5**, **Table 4**).

**Table 4:** The mechanism of NSUN2 in malignant tumors.

Malignant tumors types	Roles	Mechanism (target/signaling pathway)	References
breast cancer	tumor promoter	HGH1	(186)
lung cancer	tumor promoter	ME1/GLUT3/CDK2	(47)
lung adenocarcinoma	tumor promoter	PIK3R2/PI3K-AKT	(187)
HCC	tumor promoter	PKM2	(48)
HCC	tumor promoter	Wnt	(188)
CRC	tumor promoter	YBX1/M5C-ENO1	(190)
CRC	tumor promoter	SKIL	(191)
OS	tumor promoter	FABP5	(192)

#### 2.2.2 Others

Numerous m5C modification writers are implicated in the progression of malignant tumors and offer potential therapeutic insights (**Table 5**). For instance, knocking down NSUN3 increases the infiltration of M1 macrophages while reducing M2 macrophages, thereby promoting the progression of head and neck squamous cell carcinoma (193). In CRC, NSUN3 knockdown activates AMPK-related signaling and inhibits STAT3 signaling, leading to anti-phosphorylation and pro-apoptotic effects, which suppress CRC cell proliferation and migration (194). These findings suggest that NSUN3 inhibitors could serve as a novel therapeutic strategy for malignant tumors. NSUN4 mediates m5C modification of Circacteri3, which targets DNA binding protein 1 (DDB1), affecting mitochondrial function and energy metabolism, and thereby promoting lung cancer development (195). In gliomas, NSUN4 drives malignant progression by increasing the stability of CDC42 mRNA (196). Additionally, NSUN4 exacerbates liver cancer and may serve as a new prognostic marker for liver cancer (197). NSUN5 promotes HCC cell proliferation through the ZBED3/WNT/β-catenin signaling pathway (198). It also recruits WDR5 to enrich trimethylated histone H3 at lysine 4 (H3K4me3) in the SMAD3 promoter region, where SMAD3 mediates the EMT pathway, further promoting HCC progression (199). In GC, NSUN5 activates the Wnt/β-catenin signaling pathway, promoting immune escape and malignant progression (200). Moreover, NSUN5 directly binds to METTL1 transcripts, enhancing their m5C modification and driving the malignant progression of esophageal cancer (201). In glioblastoma (GBM), NSUN5 enhances protein synthesis necessary for tumor progression, thereby promoting malignancy (202). In lung cancer, NSUN6 regulates the expression of NM23-H1, promoting cancer cell proliferation, migration, and EMT (203). In COAD cells, NSUN6 up-regulates METTL3 expression and mediates its m5C modification, facilitating cancer progression (204). Additionally, NSUN6 promotes m5C modification of NDRG1 mRNA, driving resistance to radiotherapy in cervical cancer (205). Targeting NSUN6 through these mechanisms may represent a promising approach to improve the poor prognosis of malignant tumors. Lastly, DNMT2 targets the pro-apoptotic gene TNFSF10 (TRAIL), promoting the proliferation of hepatocellular carcinoma cells (206). The above mechanism indicates that different m5C writers play highly specialized roles in tumorigenesis by regulating the fate of specific RNAs. NSUN4 can affect energy metabolism through mitochondrial associated RNA (such as circERI3), revealing the unique function of m5C at the subcellular level. NSUN3 and NSUN5 have begun to demonstrate their ability to regulate the tumor immune microenvironment, affecting macrophage polarization and T cell function, providing new targets for combined immunotherapy. In the future, it is necessary to continue exploring effective strategies targeting m5C modification to reverse tumor metabolic abnormalities and overcome immune resistance (**Table 5**).

**Table 5:** The mechanism of other m5C writers in malignant tumors.

Malignant tumors types	RNA-modifying proteins	Roles	Mechanism(target/signaling pathway)	References
head and neck squamous cell carcinoma	NSUN3	tumor promoter	M1/M2	(193)
CRC	NSUN3	tumor suppressor	AMPK/STAT3	(194)
lung cancer	NSUN4	tumor promoter	DDB1	(195)
gliomas	NSUN4	tumor promoter	CDC42	(196)
HCC	NSUN5	tumor promoter	ZBED3/WNT/β-catenin	(198)
HCC	NSUN5	tumor promoter	WDR5/SMAD/H3K4me3/EMT	(199)
GC	NSUN5	tumor promoter	Wnt/β-catenin	(200)
esophageal cancer	NSUN5	tumor promoter	METTL1	(201)
lung cancer	NSUN6	tumor promoter	NM23-H1	(203)
COAD	NSUN6	tumor promoter	METTL3	(204)
cervical cancer	NSUN6	tumor promoter	NDRG1	(205)
hepatocellular carcinoma	DNMT2	tumor promoter	TNFSF10	(206)

### 2.3 m1A

M1A modification is an emerging but highly promising field in the tumor epigenetic transcriptome. There is not much research on the mechanism of m1A writer in tumors, and currently only the following studies provide new strategies for the treatment of malignant tumors. TRMT6 promotes cell proliferation in HCC through the PI3K/AKT signaling pathway, providing direction for the treatment of HCC (49). In addition, TRMT6/TRMT61A can drive cholesterol synthesis to activate hedgehog signaling, promoting the occurrence of HCC (50). Another type of m1A writer TRMT10C can promote the malignant development of certain gynecological cancers and may become an effective target for treating gynecological cancers (207). Overall, m1A modification finely regulates protein translation by modifying tRNA, thereby affecting key oncogenic pathways. M1A modification may not directly cause drastic transcriptome changes, but rather exert an impact by optimizing the synthesis efficiency of specific oncogenic proteins, acting as a “translation level” amplifier. In the future, we can further explore the modification of m1A on ribosomal RNA (rRNA) and its regulation of the translation machine’s own function. Based on the discovered TRMT6/TRMT61A small molecule inhibitors, we can promote their translation into preclinical therapeutic research.

### 2.4 m7G

**Figure 6:**
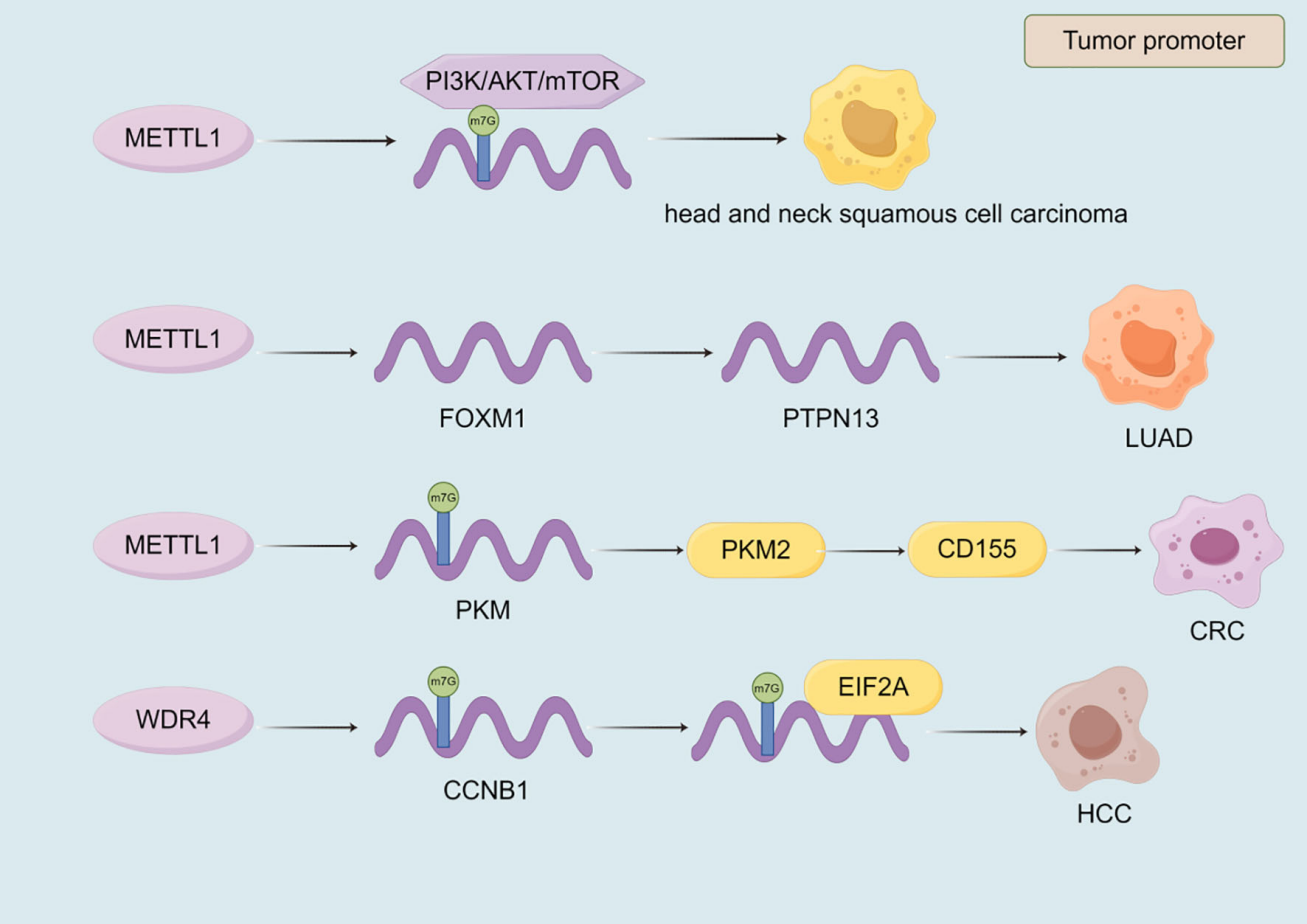
The role of m7G writers in the progression of malignant tumors. Most of the m7G writers currently discovered have a promoting effect in malignant tumors. METTL1 and WDR4 affect the progression of malignant tumors by regulating the expression of target genes, transcriptional stability, and signaling pathways.

METTL1 plays a significant role in the progression of various cancers by regulating key signaling pathways, mRNA translation, and immune modulation (**Table 6**, **Figure 6**). In head and neck squamous cell carcinoma, METTL1 promotes tumor progression by regulating the PI3K/AKT/mTOR signaling pathway, enhancing mRNA translation, and influencing the immune microenvironment (208). Conversely, in breast cancer, METTL1 mediates m7G modification to regulate cell cycle arrest and the translation of genes such as GADD45A and RB1, thereby inhibiting cancer cell proliferation (209). In LUAD, METTL1 enhances the RNA stability of FOXM1 and upregulates its expression. FOXM1, in turn, inhibits PTPN13 expression, reducing the sensitivity of LUAD cells to gefitinib and driving cancer progression (210). In CRC, METTL1 mediates m7G methylation of PKM mRNA, enhancing PKM2 expression. PKM2 activates CD155 expression, inducing immune evasion and promoting CRC progression, which provides a potential direction for CRC immunotherapy (211). Additionally, METTL1 and WDR4 collaborate to promote the malignant progression of lung cancer by mediating m7G tRNA modification and regulating mRNA codon composition to enhance mRNA translation (51). In HCC, WDR4 interacts with METTL1 to synergistically regulate m7G mRNA modification, thereby promoting cancer progression (212). WDR4 also enhances the stability of CCNB1 mRNA and facilitates the binding of EIF2A to CCNB1 mRNA, enhancing CCNB1 translation and driving HCC progression (213). Similarly, in intrahepatic cholangiocarcinoma, METTL1 and WDR4 mediate m7G tRNA modification to enhance mRNA translation, promoting cancer progression and contributing to poor prognosis (42). In osteosarcoma, METTL1 and WDR4 mediate m7G modification of tRNA, enhancing mRNA translation and promoting cancer cell proliferation, migration, and chemoresistance (52). In summary, m7G modification globally reshapes the proteome at the translation level by altering tRNA abundance and codon preference, thereby simultaneously coordinating the cell cycle, driving chemotherapy resistance, and promoting immune escape (**Figure 6**, **Table 6**).

**Table 6:** The mechanism of m7G writers in malignant tumors.

Malignant tumors types	RNA-modifying proteins	Roles	Mechanism(target/signaling pathway)	References
head and neck squamous cell carcinoma	METTL1	tumor promoter	PI3K/AKT/ mTOR	(208)
breast cancer	METTL1	tumor suppressor	GADD45A/RB1	(209)
lung adenocarcinoma	METTL1	tumor promoter	FOXM1	(210)
CRC	METTL1	tumor promoter	PKM/PKM2/ CD155	(211)
HCC	WDR4	tumor promoter	CCNB1/EIF2A	(213)

## 3 Potential clinical treatment of malignant tumors by RNA methylation modified writers

**Figure 7:**
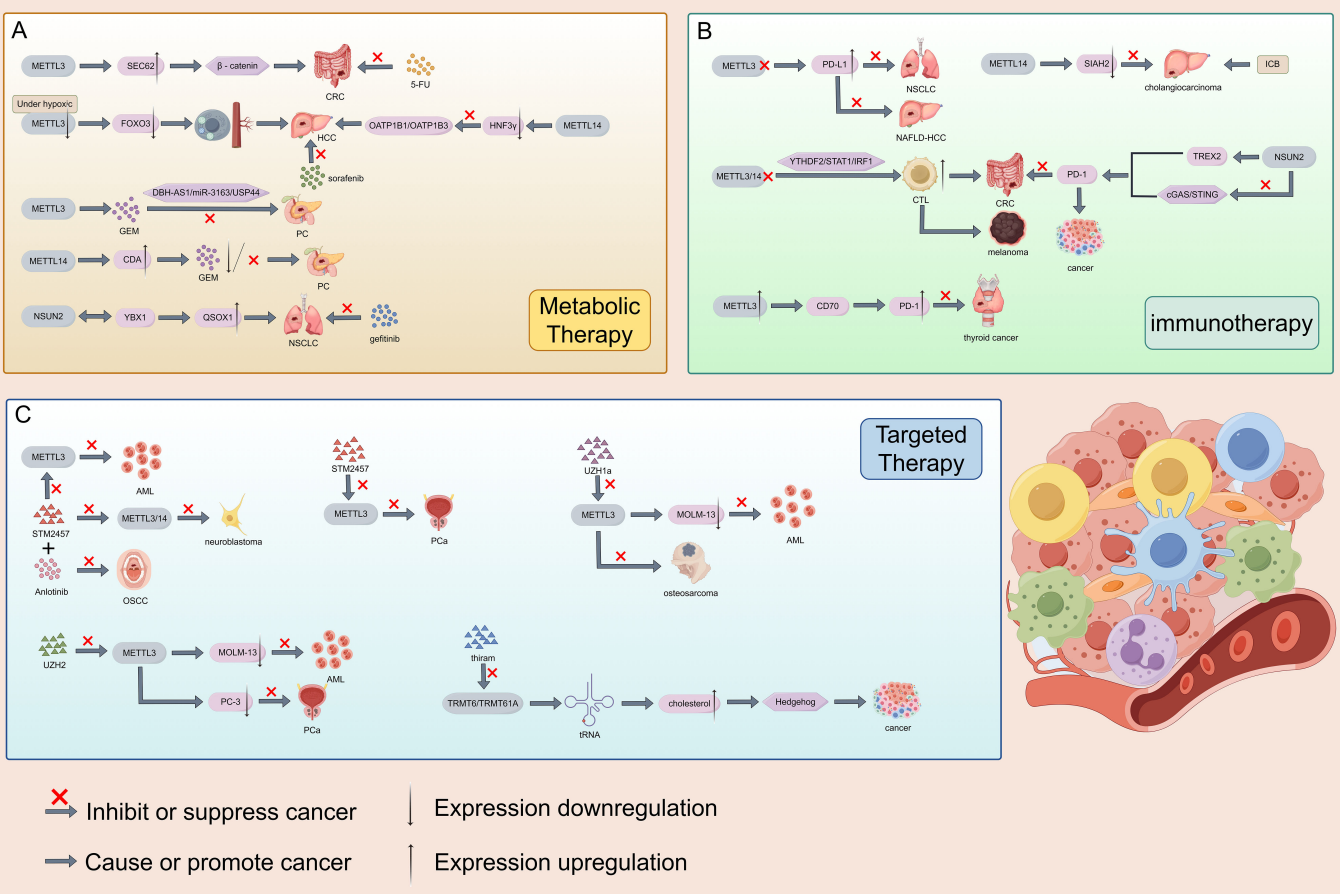
Clinical study on the treatment of malignant tumors using RNA methylation modified writers. There are currently three types of therapies, namely metabolic therapy, immunotherapy, and targeted therapy. Preliminary studies have shown that it is effective for malignant tumors such as CRC, HCC, and NSCLC.

Based on the impact and molecular mechanisms of RNA methylation modification on malignant tumors mentioned above, clinical research on the treatment of malignant tumors targeting RNA methylation modification authors is becoming increasingly popular. At present, researchers are exploring the treatment of various malignant tumors related to RNA methylation modification in clinical practice through the development of metabolic therapy, immunotherapy, and targeted therapy. RNA methylation modification writer is currently involved in regulating the resistance of various malignant tumor drugs. METTL3 can promote CRC resistance to 5-FU by activating the β - catenin signaling pathway through the promotion of SEC62 protein expression (214). Under hypoxic conditions, METTL3 deficiency leads to a decrease in FOXO3 expression, promoting autophagy and angiogenesis, thereby promoting the progression of HCC and sorafenib resistance (215). On the contrary, METTL3, as a sensitizer in PC, can enhance the sensitivity of gemcitabin (GEM) and achieve anti-cancer effects through the DBH-AS1/miR-3163/USP44 pathway (216). In HCC, METTL14 can reduce hepatocyte nuclear factor 3- γ (HNF3γ) and inhibit the activation of drug transporters OATP1B1/OATP1B3, leading to resistance to sorafenib (217). In addition, METTL14, as a resistance factor, can upregulate the expression of cytidine deaminase (CDA) to metabolize and inactivate GEM, promoting the progression of PC (218). In NSCLC, the interaction between m5C writer NSUN2 and YBX1 can enhance the translation of QSOX1 mRNA and promote intrinsic resistance to gefitinib (219). The m6A writer is a key regulator of tumor immune therapy response, particularly in immune checkpoint blockade therapy (ICB) and adoptive cell therapy (ACT). PD-L1 is a key protein used by tumor cells to suppress T cells and achieve immune escape. In the vast majority of cases, inhibiting METTL3 can enhance the efficacy of anti-PD-1 therapy. For example, knocking out METTL3 in NSCLC and non-alcoholic fatty liver cancer-related liver cancer (NAFLD-HCC) increases PD-L1 expression and immune cell infiltration, ultimately enhancing the anti-PD-1 effect and achieving anti-tumor efficacy (220, 221). Knocking down METTL3/14 enhances the infiltration and function of cytotoxic T cells (CTLs) through the YTHDF2/STAT1/IRF1 axis, thereby enhancing the anti-PD-1 efficacy of CRC, which also has the same effect in melanoma (222). On the contrary, in certain situations, high expression of METTL3 is beneficial for immunotherapy. For example, in thyroid cancer, high expression of METTL3 inhibits CD70 and maintains its degradation, instead relieving the inhibition of T cells and enhancing PD-1 efficacy (223). The same effect is also observed in macrophages of melanoma and lung cancer (224). In addition, METTL14 may downregulate the expression of SIAH2, making cholangiocarcinoma sensitive to ICB (225). There is relatively little research on the clinical treatment of malignant tumors with ACT, and further exploration is needed. At present, progress has shown that METTL3 can enhance the proliferation and cytotoxicity of NK cells, providing direction for future research (226). In addition, due to the correlation between m6A modified regulatory factors and T cell function, CAR-T cell therapy is also worth looking forward to (227, 228). Researchers have studied the inhibitory effect of m6A writers on certain malignant tumors in the development of targeted agents. Li et al. utilized the protein-protein interaction at the METTL3-METTL14 binding interface to select a candidate peptide RM3 with high anti-cancer activity, and then designed a fixed peptide inhibitor (RSM3) to enhance the stability of RM3, thereby achieving the anti-cancer effect of targeting carcinogenic METTL3 (229). Small molecule inhibition is an important anti-cancer strategy. STM2457, as a highly effective catalytic inhibitor, targets METTL3 to promote AML apoptosis (230). In addition, STM2457 can also inhibit the progression of PCa (231) and neuroblastoma (232). STM2457 can provide new insights into the treatment of OSCC in conjunction with Anlotinib (233). There are two other METTL3 inhibitors that can also be effective in clinical treatment. UZH1a has been shown to reduce m6A/A levels in the mRNA component of leukemia cell line MOLM-13, thereby inhibiting tumor cell proliferation and exhibiting the same effect in osteosarcoma U2OS cells (234). UZH2 can also reduce the m6A/A levels of polyadenylation RNA in MOLM-13 (acute myeloid leukemia) and PC-3 (prostate cancer) cell lines (235). In addition to m6A writers’ research on the clinical treatment of malignant tumors, other RNA methylation modifications also provide direction for the clinical treatment of malignant tumors. M5C writer NSUN2 can regulate the expression of TREX2 and inhibit the cGAS/STING pathway, thereby developing resistance to PD-1 blockade and providing new ideas for immunotherapy (236). In addition, researchers have identified a potent inhibitor thiram for m1A writer TRMT6/TRMT61A, which exhibits significant therapeutic effects in liver cancer treatment. Thiram inhibits TRMT6/TRMT61A in liver cancer, increases the methylation level of some tRNAs, triggers cholesterol synthesis, activates the Hedgehog signaling pathway, and promotes the process of liver cancer formation (50). At present, there is not much research on other RNA writer inhibitors, and targeting RNA methylation modified writers with inhibitors for malignant tumor treatment may become the most direct and convenient direction. However, there are still several issues to be addressed on the future research path. Firstly, the actual effect of using inhibitors to treat patients is unknown, and currently it can only be the direction of treatment rather than the exact treatment method. Secondly, patients with malignant tumors may gradually develop resistance during the treatment with RNA methylation modified writer inhibitors, which cannot guarantee the expected therapeutic effect. Thirdly, due to individual differences in constitution, inhibitor drugs are not suitable for every patient with malignant tumors, which may narrow the scope of treatment. Finally, the selection of inhibitors also needs further evaluation to discover their potential effects. In addition, according to the above report, the promotion or blockade of targeted genes and signaling pathways within the mechanism can also become the direction of future treatment, but more detailed and lengthy research is needed, and the task is arduous and the road is long (**Figure 7**).

## 4 Conclusions and outlook

In this review, we mainly introduce and summarize common RNA writers and their mechanisms of action in different malignant tumors, providing direction for the treatment of malignant tumors. Firstly, in many studies, we have found that m6A writers have a dual role in the progression of malignant tumors, that is, m6A writers can promote or inhibit the progression of malignant tumors, and the mechanisms exhibited in different malignant tumors are different. For example, METTL3 can promote the progress of esophageal cancer, colorectal cancer, pancreatic cancer and other cancers, and the mechanisms of promoting the occurrence of different targets among them are also different. More interestingly, the same m6A writer within the same malignant tumor can regulate tumor progression through different mechanisms, providing diverse targeted therapies for cancer patients as much as possible. For example, in cervical cancer, we identified three different mechanisms of METTL3 that can promote the progression of cervical cancer. HSPA9 (68), NEK2 (69), and SLC38A1 (70) are the targets corresponding to these three mechanisms and may become effective targets for cancer treatment in the future. We also found that in some studies, the same m6A writer can have opposite effects on the same cancer, such as METTL3 promoting non-small cell lung cancer (84), while also inhibiting lung cancer cell migration and suppressing lung adenocarcinoma progression (86, 87). In addition, there have been many reports on the relationship between m6A writers and long non coding RNAs in the study of their impact on the progression of malignant tumors. For example, METTL3 can promote the development of PTC by increasing the stability of LINC00894 (61), and METTL14 mediated lncRNA RP1-228H13.5 can promote the development of liver cancer (95). However, there are few reports on research related to circular RNA, only in colorectal cancer, prostate cancer, glioma, lung adenocarcinoma, and cervical cancer. Finally, m6A writer plays a role in immunotherapy and drug-resistant therapy for malignant tumors. WTAP can enhance the immune escape mechanism and promote the occurrence of colorectal cancer (123). KIAA1429 can reduce the sensitivity of liver cancer cells to the chemotherapy drug sorafenib and enhance the resistance of gastric cancer cells to oxaliplatin chemotherapy, thereby affecting the development of cancer (237). The research on m6A writers can help us explore the occurrence of malignant tumors and provide new directions for the treatment of malignant tumors. Currently, there have been breakthroughs in the treatment direction of METTL3 inhibitors, but other aspects of research have not yet been discovered. The road to a comprehensive understanding of the relationship between m6A writers and malignant tumors is still long. In addition, the mechanisms of action of m5C, m1A, and m7G modified writers in malignant tumors have also been gradually studied, providing more possibilities for the treatment of malignant tumors. We found that immunotherapy may play a better role in exploring the treatment of m5C modified writers. For example, in head and neck squamous cell carcinoma, it acts on M1 and M2 macrophages by affecting NSUN3 (193). NSUN5 can promote immune escape in GC (200). At present, there is a lack of research on writer inhibitors for these three modifications, but there are already mechanism directions that provide inspiration. Meanwhile, emerging treatment approaches such as mRNA therapy also require RNA methylation modifications to enhance stability and avoid immunogenicity (237). Despite significant progress in research, there are still many challenges in this field: the functional background of writers is dependent, and the same writer (such as METTL3, METTL14) can play completely opposite roles in different tumors or even within the same tumor. The determining factors of upstream regulation and downstream substrate selectivity are still unclear. Writers have targeted specificity and toxicity, and they typically modify a large number of RNA transcripts. How to develop drugs that can specifically block carcinogenic modification events without interfering with global RNA homeostasis and normal physiological functions is a major challenge. Similar to other targeted therapies, tumors may eventually develop resistance to writer inhibitors. Is combination therapy strategy (such as combining with immune checkpoint inhibitors) an inevitable choice? This issue also needs to be considered. Although small molecule inhibitors such as STM2457 have been studied, their *in vivo* delivery efficiency, tumor targeting, and long-term safety still need to be further evaluated. Our research on circRNA is insufficient. Compared with lncRNA, the study of how writers affect tumor progression by regulating circular RNA is still in its infancy, which is a huge potential unknown field. Based on the above summary and challenges, we believe that future research should prioritize deeper mechanism exploration: using single-cell multi omics techniques to draw RNA modification profiles in the tumor microenvironment, in order to analyze the cellular background specificity of writer function. In addition, it is necessary to develop new treatment strategies and explore combination therapies, such as combining writer inhibitors with chemotherapy, radiotherapy, immunotherapy, or metabolic drugs, to overcome drug resistance. Develop new modal drugs such as protein degradation targeted chimeras to achieve more thorough and precise clearance of writer proteins. Strengthen clinical translation and promote the clinical validation of writers or their modified signatures as predictive biomarkers to guide patient stratification and treatment selection. Actively exploring the synergistic effect of mRNA based therapy and RNA methylation modification, utilizing modification techniques to enhance the stability and translation efficiency of therapeutic mRNA. In summary, RNA methylation writers are a dynamic but extremely complex link in the cancer regulatory network. Looking ahead to the future, we hope to discover more types of m6A writers, gain a deeper understanding of the structures and mechanisms of existing molecules, closely integrate epigenetic transcriptomics, functional genomics, and clinical data, develop diversified targeted therapies for different cancers, and provide more support and choices for patients with malignant tumors.
